# User Perspectives of Characteristics of Improved Cookstoves from a Field Evaluation in Western Kenya

**DOI:** 10.3390/ijerph13020167

**Published:** 2016-01-27

**Authors:** Jennifer D. Loo, Lirije Hyseni, Rosebel Ouda, Selline Koske, Ronald Nyagol, Ibrahim Sadumah, Michelle Bashin, Mike Sage, Nigel Bruce, Tamara Pilishvili, Debbi Stanistreet

**Affiliations:** 1 Respiratory Diseases Branch, National Center for Immunizations and Respiratory Diseases, Centers for Disease Control and Prevention, 1600 Clifton Road NE, Mailstop C-25, Atlanta, GA 30329, USA; tdp4@cdc.gov; 2 Department of Public Health and Policy, Institute of Psychology, Health and Society, University of Liverpool, Liverpool L69 3GB, UK; L.Hyseni@liverpool.ac.uk (L.H.); ngb@liverpool.ac.uk (N.B.); D.L.Stanistreet@liverpool.ac.uk (D.S.); 3 Research Unit, Safe Water and AIDS Project, Kisumu 40100, Kenya; rozzypats@gmail.com (R.O.); chepkwemoiselin@yahoo.com (S.K.); ronotien0@yahoo.com (R.N.); sirahimahs@gmail.com (I.S.); 4 Public Health Institute, Oakland, CA 94607, USA; mbashin@phi.org; 5 Global Alliance for Clean Cookstoves, Washington, DC 20006, USA; mikesage44@gmail.com

**Keywords:** indoor air pollution, cookstoves, qualitative, field evaluations

## Abstract

Over half of the world’s population uses biomass fuels; these households cook on open fires indoors, increasing their risk of adverse health effects due to household air pollution (HAP) from biomass combustion. This study evaluated six improved cookstoves (ICS) for effectiveness and acceptability in a rural community in Western Kenya. This paper describes women’s views on each ICS compared to the traditional three-stone fire. Views on stove characteristics, fuel consumption, health effects and acceptability were assessed through structured interviews and focus group discussions. Data were coded and analyzed using a thematic approach. In total, 262 interviews and 11 focus groups were conducted from 43 women. Overall, women preferred the ICS over the traditional three-stone fire for various reasons including ease of use, efficiency, fuel efficiency and perceived reduction in smoke and improved health. However, there were clear preferences for specific ICS with almost half of women preferring a Philips stove. Despite acceptance and use of ICS, women used multiple stoves to meet their daily needs. Qualitative studies are essential to field evaluations to provide insight into user perspectives and acceptability of ICS and to inform research and development of technologies that are both effective in reducing HAP and practical in use.

## 1. Introduction

Over half of the world’s population uses biomass fuels (e.g., wood, dung, crop residues, charcoal) for cooking, the majority of whom reside in low- and middle- income countries [1]. Many of these households cook on open fires indoors, often in spaces with little ventilation, increasing their risk of adverse health effects due to household air pollution (HAP) from biomass combustion. It is estimated that more than 3.5 million deaths occur annually from HAP [2]. Women and children are often the most affected as women are the primary cooks as well as the caretakers of young children. Furthermore, women and children often bear the additional burden of collecting biomass for daily use, hindering their opportunities for education and development [3]. Finally, biomass fuel collection is unsustainable in the longer term and leads to further deforestation and climate change issues [4].

In September 2010, the Global Alliance for Clean Cookstoves (GACC), a global public-private initiative was formed with the aim to save lives and improve livelihoods by creating a thriving and unhindered global market for cleaner and more efficient cooking solutions in the developing world [5]. This initiative has since provided momentum to advance the field of cookstove technology. In more developed or urban communities, liquid petroleum gas (LPG) and other forms of clean cooking are being promoted; but, in most rural or impoverished communities, the transition to LPG or electricity has been slower and requires attention to price and supply issues. Therefore, improved biomass cookstoves (ICS), which aim to achieve higher standards of clean cooking and relieve the burden of fuel collection and use, will continue to be an important part of the solution for the meantime [6].

The majority of research in the field to date has used quantitative methodologies to measure exposure to HAP comparing ICS to traditional stoves or three-stone fires. However, quantitative studies alone rarely explore the perspective of the user [7]. Qualitative approaches are essential in field evaluations to provide insight into user perspectives and acceptability of ICS and to inform research and development of technologies that are both effective in reducing HAP and practical in use. Fewer studies have utilized in-depth qualitative methods within the study design. This methodology afforded the opportunity to describe in-depth stove use from a user perspective.

This pre-/post- single-arm intervention study evaluated six different ICS for effectiveness and acceptability in a rural community in Western Kenya. The study design is novel because it allowed the study participants to use six different ICS in their own households and compare these stoves to each other as well as to the traditional three-stone fire. An overview of the study and findings focusing on the effectiveness of ICS are reported elsewhere [8,9]. This paper describes women’s views on each ICS compared to the traditional three-stone fire as well as their perspectives when comparing an ICS to the other cookstoves tested.

## 2. Experimental Section

### 2.1. Study Design Overview

The study focused on field-testing stoves for short-term acceptability and performance in a household setting by employing a cross-over design, which allowed evaluation of several stoves within each household for two weeks per stove with a one-week break in between. The study utilized a cross-over design in order to limit the inter-household variability in HAP levels related to individual household practices, size of the household, and ventilation due to house structure. Women’s views on stove characteristics, including ease of use, fuel consumption, health effects and acceptability were assessed through structured interviews and focus group discussions. A full description of the overall study design has been detailed elsewhere [9].

### 2.2. Study Population and Sampling Procedures

Nyando District is located in Kisumu County in Western Kenya, near Lake Victoria and the city of Kisumu. There are approximately 1.1 million people residing in this area; 17% are children <5 years of age. Most households are in communities that are considered rural and the majority of households rely on subsistence farming. Eighty-six percent of households in the area are in Kenya’s poorest socio-economic quintile [10] and the majority (95%) use firewood as the main fuel for cooking [11]. Common cooking practices include making ugali, a starchy staple dish of Kenyan households that is prepared by vigorous stirring, and nyoyo, a traditional dish of corn and beans that is slow cooked.

Households using traditional three-stone fires as the primary means of cooking, having women of childbearing age (15–49 years of age) and at least one child less than 5 years of age residing in the household were eligible to participate. All households in each village were identified through a census by the village chiefs. Forty-three households were randomly selected from the list of all eligible households in the two participating villages of Nyando District. Field workers from a local non-governmental group, Safe Water and AIDS Project (SWAP), who were trained in qualitative data collection, made home visits to all selected households to conduct baseline and follow-up interviews with the primary cook to assess acceptability of the new stoves.

### 2.3. Overview of Improved Cookstoves

Six different ICS were chosen for inclusion in this field evaluation: two improved rocket stoves (Ecozoom and Envirofit), two electric fan-assisted gasifier stoves with solar panels (Philips and Ecochula), one double pot rocket with chimney stove (Prakti), and one built-in clay rocket stove with thermal-powered fan (Rocket Thermoelectric Cookstove Add-on (TECA)). These ICS were chosen based on performance in U.S. Environmental Protection Agency laboratory testing and initial assessment of acceptability using a participatory cooking demonstration in the study area (unpublished data) [12]. A more detailed description of each stove can be found in Table S1.

### 2.4. Training of Field Staff

Field staff were trained in qualitative methods by a senior behavioral scientist. A multi-day training was conducted which addressed the correct way to: (1) conduct in-depth interviews and Focus Group Discussions; (2) execute note taking and complete checklists; and (3) translate and transcribe data for the qualitative team.

Institutional review boards (IRB) at the Kenya Medical Research Institute (Protocol SSC2075) and the U.S. Centers for Disease Control and Prevention (CDC) (Protocol #6155) reviewed and approved the protocol. Written informed consent was obtained from all participating households.

### 2.5. Data Collection

Prior to stove installation, the woman primarily responsible for cooking in the household completed a brief baseline questionnaire on current stove use, cooking practices, fuel collection and consumption, and household demographics.

Improved cookstoves were placed in the 43 study households. Utilizing a cross-over study design, households tested up to six stoves for two weeks per stove with a one-week break in between. Due to logistical issues, some stoves only became available at a later period during the study; as available, stoves were randomly assigned an order in which they were tested in each household. Field workers used a standardized guide to train women to use each new stove. Their traditional stove remained in their home, but they were encouraged to use the ICS for their daily cooking. At the conclusion of each two week observation period (round), field workers again completed follow-up questionnaires, activity diaries and in-depth interviews with each cook. Focus Group Discussions were conducted partway through the study (following Round 4) and at its conclusion. In these group discussions, women provided their perspectives on stove use and function, acceptability and reasons behind multiple stove use. Additional topics included gender, access to money and affordability, the role of husbands in the decision making process, priorities, familiarity, and stove promotion; these findings have been reported elsewhere [13]. Stove use monitors were also placed on each stove in the household to track use during the study period, results for which are reported separately [14].

All in-depth interviews and focus groups were audio-recorded, translated from DhoLuo into English and transcribed by a trained member of the field work team.

### 2.6. Data Analysis

In total, 262 interviews and 11 focus groups were conducted. The data were coded and analyzed using Dedoose software (SocioCultural Research Consultants© 2014) and a thematic approach. Braun and Clarke’s (2006) six phases of analysis were used as a framework [15]. In phase one, the transcripts were read carefully to gain familiarity with the data. In phase two, initial codes were generated by coding the transcripts individually; final coding decisions were made jointly by the research team after comparing individual codes. Following this, themes were developed and reviewed by the research team (phases three and four). Finally, analytical themes were developed (phase five) and the report was written (phase 6).

All stages used established principles for analyzing qualitative data. Recording of the process of theme development was explicit to ensure the methodology was both transparent and rigorous. All interviews were coded and analyzed, initially by round in order to identify any changes to participant responses over time. Some codes were stove specific, and were recorded as such where appropriate. The descriptive themes developed fell under the broad headings of characteristics, fuel issues, and health (see Table S2).

Data from each round were coded for each stove and each theme and analyzed to the point of saturation [16]. All transcripts were carefully reviewed, but when codes became saturated (*i.e.*, no new or novel data were found), data were analyzed only where new information was offered. In practice, this differed by stove, as some stoves were introduced only in the later rounds. In the study’s first two rounds, only the Ecozoom, Envirofit, Philips and Prakti were used. During the third round, another stove was added: the Rocket TECA, and in the fourth round, the Ecochula. Thus, in the fourth, fifth and sixth round, data were mostly dominated by the Rocket TECA and the Ecochula.

Ranking of ICS from the Focus Group Discussions was calculated by assigning two points to each woman’s first choice stove and one point for each woman’s second choice stove. Points were summed for each stove and absolute point values are presented.

The percentage of multiple stove use was calculated as the number of cooking events in a 48-hour monitoring period where more than one stove was used or where a stove other than the ICS under evaluation was used divided by the total number of cooking events that occurred during the monitoring period, multiplied by 100.

## 3. Results

In total, 45 households participated in the study: 7 households received all 6 of the study stoves, 30 received 5 stoves, and the remaining 8 households received 2–4 stoves [9]. Forty-five women participated in the study (mean age 28.3 years). Eighty-eight per cent of the women were married. Eighty-four per cent either completed or had some primary education. One-third were subsistence farmers, 21% owned their own business, and 12% ran the household. The average size of households was 6 people, with 1.9 children <5 years of age. All households had access to drinking water (either from a pump, well, communal standpipe, or collected from the river) and 72% had a latrine in the yard. The average weekly expenditure per household was 1381 Kenya schillings (ksh) (~$15 USD). Over 50% of households had at least one of these items: a radio, bicycle, cell phone or cow. At baseline, all households in the study used a traditional three-stone fire as their primary stove for cooking, with half also using the stove for heating of the household. All three-stone fires were located within the house.

Based on in-depth interviews, in general, study participants reported that they enjoyed using the ICS more than the traditional three-stone fire. Women often cited efficiency, ease of use, appearance, safety, perceived health improvements and less smoke as reasons for this preference.

### 3.1. All ICS v. Traditional Three-Stone Fire

#### 3.1.1. Ease of Use

Overall, women found the ICS easier to use than the traditional three-stone fire. The majority of women found that the ICS were easy to light, especially the Philips. A few women expressed concern that the Ecozoom, Ecochula and Rocket TECA were difficult to light. Adjusting the heat was described as easy for most of the ICS, with women saying they simply added or removed firewood:

“*Adjusting the fire is easy because after adjusting it I can go back and sit down and the fire goes on well and when I see the fire is almost going off, I add more firewood.*”

(Participant Village K, Round 1)

A few difficulties were described, including that the combustion chambers filled easily with ash and had to be emptied before firewood could be added:

*“…When the wood has burnt it (the ICS) gets full with ash, so it is hard to add (wood) because it (the combustion chamber) is squeezed.”* 

(Participant Village W, Round 5)

With regard to retaining a flame and supervision of the fire during cooking, women felt that the amount of supervision required was related to the size of the fuel opening. ICS that utilized small pieces of firewood (e.g., Philips and Ecochula) required constant supervision to prevent the flame from going out. With the three-stone fire, women were able to use large pieces of hard wood and leave the stove and do other chores, only checking the stove from time to time. Views on cooking speed varied depending on the ICS. Women’s opinions on pot stability also varied depending on the ICS and the pot used for cooking.

In comparison to the traditional three-stone fire, women found most of the ICS occupied a smaller space in their kitchen and felt that they fit well within the kitchen space, although women did note that the Prakti must be strategically placed in the kitchen because the chimney must vent to the outside. They also mentioned that the ICS were cleaner and easier to keep pots clean than the three-stone fire:

*“These other stoves that we are using don’t have too much soot. You can touch the pot and your hands are still clean, there is no dirt.”* 

(Participant #4, Focus group 1.3)

#### 3.1.2. Use with Local Cooking Practices and Views on Aesthetics

When preparing dishes using the ICS, women gave mixed responses with the majority of women saying they could use these ICS to cook some dishes (*i.e.*, greens or porridge) but not others (*i.e.*, nyoyo (a traditional dish of corn and beans that is slow cooked) or ugali (a starchy staple dish of Kenyan households)). Women often returned to using the three-stone fire to cook these dishes:

*“When there was little time and I was in a hurry, then I could cook on the study stove and cook ugali on the three stones.”* 

(Participant #6, Focus Group 1.5)

Inability to cook a variety of dishes was one reason that multiple stove use was found with use of all ICS. Other reasons included speeding up cooking by using two stoves at the same time, especially when cooking for large numbers of people; not enough time to split firewood into small pieces for stoves that required smaller fuel size; and family members being unfamiliar with the ICS.

*“When I want to cook bigger amounts of food then I use my three stones…also when I am in a hurry and I want to go faster, then I will use the three stones and this new stove.”* 

(Participant Village W, Round 3)

*“The reason why I didn’t use it (ICS) was because it needs tiny pieces of wood and I had big pieces of wood and I didn’t have time to split because I was busy. (The limited time) forced me to use the other one.”* 

(Participant Village K, Round 5)

*“…The rest of [the] family members have used the three stone because they don’t know how to use the new stove.”* 

(Participant Village K, Round 2)

Overall, women were pleased with the appearance of the ICS, often referring to them as “smart”, “well designed”, and “beautiful.” Women reported mixed responses in relation to comfort when using ICS, perhaps because women had to bend low to see the fire burning or add wood to the fire.

#### 3.1.3. Functionality

All ICS were regarded as efficient compared to the three-stone fire in terms of less fuel consumption and retaining and directing heat. Functionally, women generally felt that the combustion chamber was too small as they were constantly removing ash from the chamber to keep the fire going. Satisfaction with the size of the fuel opening varied by stove type and women preferred ICS with larger openings for fuel. In terms of maintenance and durability, the main issue with ICS that had electronic parts was the availability of parts such as wires and batteries if broken or worn out. There was a particular concern with the Philips and Ecochula of how to fix the solar panel if it broke:

*“... There are times when it[Philips] stops functioning and ... there is no one who can repair it. (A broken part) could force me to use three stone until it is repaired.”* 

(Participant #2, Focus group 2.1)

#### 3.1.4. Fuel Issues

All women reported a perceived use of less firewood for cooking, regardless of the stove type. There was no single stove that they thought reduced firewood use more than others. Data regarding fuel availability and fuel collection were very limited. However, women noted that, because the ICS use less firewood, they did not have to collect as much or as often as with the three-stone fire.

*“These stoves have really helped us in terms of fuel consumption … we have a problem getting fuel in our area, the fact that they use less fuel has really helped us.”* 

(Participant #1, Focus group 1.4)

In terms of quality of biomass, the only challenge mentioned was the use of wet wood as it was difficult to light and produced more smoke, which was consistent across all stoves and an issue when using the traditional three-stone as well.

*“If you have dry fuel then you don’t have any problem, but if you have wet fuel then there may be a problem and you may think they (the ICS) are slow.”* 

(Participant #7, Focus group 1.4)

#### 3.1.5. Health 

When specifically comparing health and symptoms with the three-stone fire, nearly all women indicated a preference for the ICS. No individual stove was reported as being better in this respect. The women reported that ill health symptoms such as coughing and headaches were either reduced or no longer present.

*“I have seen a great change with them (ICS) because when we were using the old ones we used to get sick most often for example flu, headache but nowadays I see they [respiratory symptoms] are reduced … it is of great help to our health.”* 

(Participant #1, Focus group 1.4)

Most women believed that this was due to smoke reduction, which was noticeable with all ICS.

*“…Our stoves were interfering with our lives. There was a lot of smoke. Sometimes you cough, chest pains, running nose when cooking until you look like one who is crying. But since you brought them (ICS) there is no smoke. That’s what I am experiencing.”* 

(Participant #3, Focus group 1.5)

Women did comment that there was smoke while initially lighting the ICS, but after this time, little or no smoke was produced. Women noted that there was smoke when the stove was overstuffed or the firewood was wet.

In relation to child health, two key themes emerged. First, women reported that children’s health improved as a result of reduced smoke:

*“…Children like sitting next to us when we are cooking and within a short time you hear them cry ‘Mother the smoke is choking me’ but nowadays you just cook and the kids just sing next to you and tell stories so we have found a great change.”* 

(Participant #1, Focus group 1.4)

Second, risk of burns continued to be a general health concern, specifically with the chimney stove.

*“The only thing I fear with it (Prakti) is just the pipe that removes smoke out of the house. You see it gets very hot. I have been thinking that something could have been put around it to cover it... the child can touch it and get burnt.”* 

(Participant Village W, Round 3)

There were limited data regarding safety. Women discussed safety in relation to their children and risk of burns; they were particularly concerned with the Prakti as it was perceived to increase burn risk due to the hot chimney. No concerns were raised over the risk of any of the ICS causing a fire in the household.

#### 3.1.6. Suggested Stove Improvements

Overall, women liked all six ICS, but Philips emerged as the strong preference by the majority of women. However, when asked about how these ICS could be improved, women noted potential improvements they would like to see in all ICS, including: enlarging the combustion chamber and fuel openings, making the cooking surface bigger for use of bigger pots, and using more durable construction materials. Concerns across most ICS included the small combustion chamber, need for constant supervision, small cooking surface and need for frequent removal of ash.

Women also suggested these stove-specific improvements (responses included if suggested by one or more woman) (Table 1).

**Table 1:** Suggested improvements of improved cookstoves.

**Ecochula**	Put stands at the bottom to prevent stove from becoming damaged while being pulled from the stand Change the way fuel is added to make adding fuel easier (such as adding fuel from the sides or top) Improve the fan to blow the fire more efficiently
**Ecozoom**	Make spaces in rack smaller (so wood does not fall through when partially burnt) Widen bottom part of stove for increased stability Make the stove lighter in weight Provide bigger handles to carry stove Make pot rest closer to combustion chamber
**Envirofit**	Include a rack to support firewood inside stove Make handles out of a material that is less slippery and does not retain heat Increase pot stability by making six stands on the pot rest Increase height of the stove Reduce spaces in rack to prevent ash from falling onto floor
**Philips**	Protect switch from damage Improve solar panel to charge cell phones Smooth ends where the pot rests (too sharp) Use material other than plastic to make the bottom of stove Increase pot stability
**Prakti**	Add a rack to support firewood Re-position chimney Add three prongs for pot to rest
**Rocket TECA**	Provide bigger combustion chamber Reduce height of stove Improve ease of lighting stove

### 3.2. Stove-Specific Findings

#### 3.2.1. Philips (Fan Stove Powered by a Solar Panel)

Women ranked the Philips stove first among all ICS evaluated. Study participants found the Philips one of the most efficient stoves due to the fast cooking speed, flames that were better directed towards the pot, and good retention of heat. Additionally, women found the Philips the easiest to use because it was easy to light and easy to adjust heat:

*“I also like Philips stove because it is faster and consumes less fuel and once you have charged it with the solar panel then the food just gets cooked within a very short time.”* 

(Participant #4, Focus Group 1.2)

*“There isn’t anything hard. Once I have put in the small pieces of fuel, it just lights very fast …I just strike the match box and light the pieces of paper and then I switch on the power button and the fire just burns.”* 

(Participant Village W, Round 3)

Women appreciated the stove’s portability, especially during the floods of the rainy season, and the small space that the stove occupied in the kitchen. Additionally, women liked the color of the stove (silver) and the solar panel that powered the fan. The cleanliness of this stove was also appreciated as it cooked clean food (free of ash) and did not make the pots dirty. Women reported using other stoves in conjunction with the Philips stove at least 34% of the time, with some women reporting multiple stove use if they had to wait for the Philips stove to charge. Some women expressed concern in using the Philips as they sometimes burned their fingers on the metal when adding firewood from the top:

“*You feed it from the top. So when you feed it, it sometimes burns your fingers.*”

( Participant Village W, Round 1)

Additionally, Philips was seen as the least durable as women raised concerns about the plastic materials, faulty switches and weak base. The bottom of the stove was made of plastic, which women feared might burn or break. Other concerns expressed included: small fuel opening; a need to get familiar with the stove’s fast cooking speed; lack of charge from the solar panel at times (either from need for charging or improper function); feasibility of repairs; and risk of burns when emptying ash while cooking.

#### 3.2.2. Rocket TECA (Traditional Clay Rocket Stove with Fan Powered by Internal Thermo-Electric Generator)

The Rocket TECA ranked second overall in preferential stove choice among users. Users appreciated the Rocket TECA for the ability to use the stove even when there is flooding (due to the stove’s height) and additional space on top of the stove that allowed for placing and storing things while cooking. Women that used the stove also noted that the stove fit well in their kitchen, the fan was efficient, the heat was easy to adjust, the stove retained heat well, both flat bottom and round bottom pots could be used for cooking, pots were stable, and dishes were easy to cook:

“*I do not see anything hard about cooking ugali there because if you place a pot there or a wok it does not slide; it is just stable.*”

(Participant Village W, Round 5)

“*The materials that are used to make the stove really retain fire and heat. You can cook and especially if you were using hard wood, then you will find that when you put something in afterwards, the heat in the stove will keep on warming the food.*”

(Participant Village K, Round 5)

“*This rocket is good, it retains heat and when it has the machine [TECA fan insert] then it is better and you don’t even blow the fire you just adjust …even that pot rest is good because if you put a pot there it fits well.*”

(Participant #1, Focus Group 2.5)

Although women ranked this stove second, there are a number of characteristics that they thought could be improved. Many women found the stove difficult to use because it was hard to light, the fan only worked after the fire was lit (this is due to the use of a thermoelectric generator) and they also found the fuel opening quite small making adding firewood difficult. In addition, it filled with ash quicker than other stoves, and the ash was sometimes hard to remove from the chamber:

“*The hard thing is the way it is deep. After the fire wood has burned to the end, you must push in the firewood to the end and you must remove the remaining ash in order to continue cooking.*”

(Participant Village K, Round 4)

Other concerns voiced by the women included: the fan did not work very well; cost to repair the stove if broken; the fire did not reach the bottom of the pot; slow cooking speed, especially when cooking for a large family; and difficulty emptying ash. At least 37% of the women used additional or alternative stoves when cooking with the Rocket TECA.

#### 3.2.3. Envirofit (Metal Rocket Stove)

Overall**,** the Envirofit rocket stove was ranked third by the women. Most women found the stove easy to use and easy to adjust the heat. Women also appreciated the portability of the stove:

“*This Envirofit is good for me because I put firewood [in the stove] and that stand holds for me the fuel and it burns so well …it cooks faster and I can carry it and cook with it in the compound or in the other house …so its work is easy for me.*”

(Participant #1, Focus group 2.5)

Additionally, women found the stove comfortable to use as they did not have to bend over when standing and thought the fuel opening was wide and spacious for use. Views on cooking speed were mixed. Women noted that they did not think the stove was efficient when there was a lot of wind coming in from outside, as the wind blew the fire, or when they were cooking for large groups of people. Additionally, some women also found the stove hard to light and complained that the fire went out if left unattended:

*“You know for it to light quickly is not very easy. When there is a lot of wind it is hard to cook on the stove because the mouth is big and so the wind blows the fire out such that the fire does not burn in one direction...”* 

(Participant Village W, Round 3)

They also noted that larger pots were not very stable and required holding during cooking:

*“When you use a bigger pot then it is going to force you to support it thoroughly while you are cooking. You know if you do not give it support then it is not stable. So you have to hold it with your hands while you are cooking.”* 

(Participant Village K, Round 3)

Regarding safety, the women voiced concerns that the fire sometimes came outside the chamber and could cause burns. Women had mixed opinions regarding cleanliness, some reporting that soot often formed on the outside of the stove and that ash fell onto the floor when the combustion chamber was full. However, they found the pots easy to clean and no ash ended up in the food. Overall concerns using the Envirofit included: slippery and hot handles, making the stove impossible to handle until cool; the lower part of stove looked like it could get worn out quickly; lighting the stove and retaining a flame was difficult; the pot rests were very sharp and could damage cooking pots; the wood needed to be balanced when putting it in the combustion chamber; and the stove could not accommodate large cooking pots, which are often needed for cooking for large groups of people. About 30% of women reported using multiple stoves with the Envirofit.

#### 3.2.4. Ecozoom (Metal Rocket Stove)

Although women ranked this stove fourth out of six, they saw many positive aspects to the Ecozoom. Like the Envirofit, women appreciated the portability of the stove, especially during the floods of the rainy season, and also preferred this stove as it cooked cleanly (*i.e.*, ash did not get into the food) and did not make the pots dirty:

*“... It is a stove that you can cook with anywhere. That is number one. Secondly, it is a stove that cooks clean and well-cooked food. There is no ash that goes to your food.”* 

(Participant Village W, Round 3)

Women also found the stove comfortable to use as they did not have to bend when standing and had the option to sit:

*“Its height is okay. It is good; you can even cook while sitting on a stool. It is very comfortable. You just sit and cook with ease. Its height is very smart.”* 

(Participant Village K, Round 3)

Most women thought the stove was efficient due to its fast cooking speed, as the flames were directly under the pot, although some women disagreed. Heat retention was good, and it used less firewood. Also, the women reported that the size of the fuel opening was sufficient. Furthermore, most women found this stove easy to use and liked the rack that held the firewood in the combustion chamber:

*“It is well designed and it is better than the other one because it has something where the fuel is placed. It is also of good length, its color is also unique [blue], and the pot also fits there well. It also has handles which can be used to hold it.”* 

(Participant Village K, Round 3)

There were mixed comments regarding heat adjustment, with some women noting that the deep combustion chamber meant that the pot took longer to heat and constant supervision was required to ensure that the fire continued to burn. Women also noted that it was not appropriate for cooking foods like ugali and nyoyo and expressed concern that round-bottom woks were not as stable when using this stove, increasing the potential risk for burns:

*“I do not cook ugali on it because when you put the wok in it, it just goes round (sliding) meaning it is not stable.”* 

(Participant Village K, Round 1)

Additionally, women reported that the combustion chamber was too small, and therefore the ash needed to be poured out frequently. Women reported multiple stove use with the Ecozoom at least 27% of the time.

#### 3.2.5. Prakti (Double Pot Rocket with Chimney)

The Prakti stove was ranked fifth in preference with stove users. Women liked that the stove had a chimney that potentially directed smoke outside. They also liked the intended versatility of two burners to allow cooking/warming of multiple items at the same time:

*“So you see I can cook two things at the same time. That is the good thing about this stove. You can cook on one side and you put some light food that cooks faster on the other side and it will just cook.”* 

(Participant Village W, Round 1)

However, they found the stove cooked slowly, with some women even saying it was slower than the traditional three-stone fire, and the combustion chamber filled quickly with ash, requiring constant emptying or ash poured onto the floor:

“*The only difficulty that I have experienced is when the fire has lit for long and you want to add the wood you will find that (the combustion chamber) is already full … you cannot remove the ash because when you remove it pours on the floor...*”

(Participant Village W, Round 2)

Women also found the stove to be less versatile as the location of the stove was confined to areas that allowed placement of the chimney to vent outside and noted that they could only use small pots for cooking as the burners were quite close together:

“*What makes it hard is that it requires a small pot. It does not allow for a big one like this one. If you use a big one, it will force you to take some iron sheet to cover the other side (burner).*”

(Participant Village K, Round 1)

The stove cooked too slowly for making food such as ugali, and they often found the stove hard to light if there was a lot of wind. While the chimney did reduce the amount of smoke inside, sometimes smoke came back into the house via the chimney. If only one burner was in use, the second burner had to be covered or smoke escaped into the house.

“*I can only see the smoke when I remove the pot from the other pot rest. That is when I can see smoke; otherwise there is no smoke at all. When I put another pot on the other pot rest then the smoke just passes through the pipe.*”

(Participant Village W, Round 2)

Users were also concerned about the potential for burns from touching the hot chimney, especially for children. Women noted use of additional stoves at least 46% of the time they used the Prakti, which was the highest proportion of multiple stove use among all ICS.

#### 3.2.6. Ecochula (Fan Stove Powered by a Solar Panel)

The Ecochula stove ranked last in preference by women in the study. Women liked the size and portability of the stove as well as the solar panel that powered the fan:

“*Using it is easy because the space it takes is small and whenever there is problem— like recently we had floods and the house was full— I could carry it and cook in a raised place. I am impressed about that.*”

(Participant Village K, Round 4)

Additionally, many noted that they could sit while cooking. However, despite finding the stove easy to use with regard to cooking speed, adjusting heat and lighting, women disliked having to constantly remove ash from the combustion chamber. Adding firewood also took a lot of effort and disrupted cooking. Most women did not like that the stand holding the pot had to be pulled out from the stove in order to add or remove wood. They felt that constantly moving the pot caused the food to cook slowly and unevenly:

“*The reason why it takes that long is because I have to pull it out and add some firewood. When you do that, the speed in which the food was cooking reduces*”

(Participant Village K, Round 4)

Women also noted more smoke when the fan was turned to the maximum setting:

“*When you switch the fan to the highest level you will find that it spreads the fire unevenly. The fire does not go in one direction.*”

(Participant Village W, Round 5)

Overall concerns included: the ability to fix the stove if broken; burns when removing ash from chamber; flames that appeared outside the stove, which could damage the wire connecting the stove to the solar panel; the stand was not stable; and it could not accommodate large pots. Women reported using additional or alternative stoves 36% of the time.

## 4. Discussion

Overall, women generally preferred the ICS over the traditional three-stone fire for various reasons including ease of use, efficiency, fuel efficiency and perceived reduction in smoke and improved health. However, there were clear preferences for specific ICS, with 44% of women preferring the Philips over any other ICS. Women preferred the Philips stove because they found it to be more efficient, cleaner and easier to use than the other ICS evaluated in the study. There were many characteristics women appreciated about the other ICS as well, including portability (Ecozoom, Ecochula and Envirofit), ability to use various pots and cook local dishes (Rocket TECA), and double burners (Prakti). Although the Philips and Ecochula are both electric fan-assisted gasifier stoves, the Ecochula was preferred least by users. Many women cited uneven cooking, difficulty in adding fuel and frequent removal of the pot from the stove as reasons.

Although women liked and used the ICS, findings showed that ICS are not a “one size fits all approach”. Women perceived advantages and disadvantages for each ICS based on how the ICS met their individual need. Stove users have different priorities and this impacts their perception and needs of a stove. Furthermore, they often used another stove in conjunction with, or in lieu of, the ICS under evaluation. When this topic was explored during Focus Group Discussions, women noted the need to cook for large groups, inability to cook local dishes with the ICS (e.g., due to cooking speed or method of cooking), and other family members’ unfamiliarity with the ICS as some of the main reasons for multiple stove use. Other studies evaluating ICS technologies have reported similar findings, noting that users often chose to use multiple stoves because each stove provided a different function for a certain cooking practice [17,18,19]. How and why women use ICS for their daily needs is necessary to take into consideration in evaluating not only the overall acceptability of the ICS, but also, the potential effect multiple stove use could have on evaluating ICS effectiveness in reducing HAP.

Although this study took place in rural Western Kenya, many of the ICS evaluated are being marketed in different cultural and socio-economic contexts and the daily cooking practices and perspectives of the women in this study were similar to that of many women in other lower- and middle- income countries [19]. In their qualitative systematic review of stove acceptability and uptake, Stanistreet *et al.* [19] found comparable results regarding stove use and acceptability, fuel savings and health. Related to time savings, they found that changes in workload varied depending on ICS and users also noted issues of having to prepare fuel (e.g., drying or chopping wood for use) or advantages of having multiple burners to use (Prakti), views similar to those of the women in this study. In regards to durability and maintenance, women expressed similar concerns as women in other studies [19]. In a study in India, which also evaluated the Philips stove, women expressed similar views to the Kenyan women in our study reporting that the Philips stove was beneficial to their daily lives and that they liked that the stove was portable, reduced smoke and fuel use, and increased cooking speed and fuel efficiency compared to their traditional cookstove [20].

The majority of studies conducted in this field thus far have focused on evaluating quantitative measures with more limited data on user perspectives; very few studies have implemented mixed methods. Our study evaluated qualitative measures, such as user perspectives, alongside quantitative measures like effectiveness of ICS in reducing HAP. Furthermore, we evaluated up to six different ICS in the same household, allowing us to standardize at least some of the factors that could affect comparisons across ICS. Qualitative methods are essential to understand what users actually value in an intervention and give increased insight into how ICS are viewed and used in real world settings. If users do not find a stove acceptable for use, no matter how effective it has been shown to be in efficacy studies in reducing emissions and HAP, they will not use it. Qualitative data can help interpret and provide better context to quantitative findings in research that utilizes mixed methods [7].

There were a few limitations to this study. While some user perspectives of the ICS were transferrable to all stoves, despite culture or geographic location, other findings were specific to the culture of Luo women and Western Kenya. Furthermore, this study was conducted in only two villages and with a young study population (*i.e.*, women of child-bearing age). Nonetheless, this study adds to the field of qualitative research focusing on ICS, but there is still a need for more qualitative studies in different settings. Secondly, we did not anticipate the significance of multiple stove use until the study was underway. Once detected, we subsequently explored this practice through additional focus groups. Additionally, the study only assessed acceptability in the short-term. Long-term acceptability has not yet been evaluated and users could change their perception of the ICS over time. However, there are plans to assess long-term acceptability of the stoves through focus group discussions. Lastly, our study provided the stoves at no cost to participants. It did not evaluate cost or marketing in terms of measuring potential uptake; however, we did evaluate user views related to cost and household decision making and have reported these findings elsewhere [13].

## 5. Conclusions

Women found all ICS evaluated to be acceptable for use, some more than others, compared to (or in addition to) their traditional three-stone fire. However, there were clear preferences for some ICS over others, and the study found that despite acceptance and use of ICS, women used multiple stoves to meet their daily needs. Qualitative studies are essential in addition to field evaluations of performance in order to provide insight into user perspectives and acceptability of ICS and to inform research and development of technologies that are both effective in reducing HAP and practical in use.

## Supplementary Materials

The following are available online at www.mdpi.com/www.mdpi.com/1660-4601/13/2/167/s1, Table S1: Description of stoves selected for the study. Table S2: Themes and codes.
