# Supplementary material for: User Perspectives of Characteristics of Improved Cookstoves from a Field Evaluation in Western Kenya
Source: Int J Environ Res Public Health. 2016 Jan 27;13(2):167. doi: 10.3390/ijerph13020167 (PMC4772187; doi:10.3390/ijerph13020167)
Supplement: Supplementary file 1 [file ijerph-13-00167-s001.pdf]

Supplementary Materials: User Perspectives of Characteristics of Improved Cookstoves from a Field Evaluation in Western Kenya

Jennifer D. Loo, Lirije Hyseni, Rosebel Ouda, Selline Koske, Ronald Nyagol, Ibrahim Sadumah, Michelle Bashin, Mike Sage, Nigel Bruce, Tamara Pilishvili and Debbi Stanistreet

Table S1. Description of stoves selected for the study.

Stove Image	Stove Name	Design	Combustion Chamber	Model Number	Manufacturer
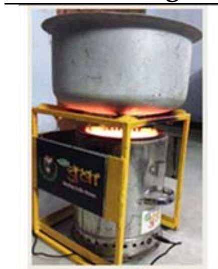	Eco Chula	Electric fan-assisted gasifier	Ceramic	XXL	Alpha Renewable Energy Pvt. Ltd. Atlanta, GA, USA
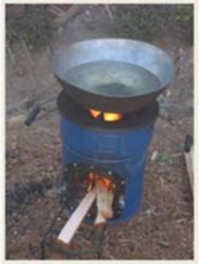	EcoZoom	Improved rocket	Ceramic	Dura	EcoZoom, OR, USA
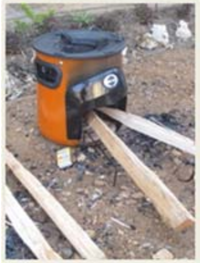	Envirofit	Improved rocket	Metal alloy	G3300	Envirofit International Fort Collins, CO, USA

Table S1. *Cont.*

Stove Image	Stove Name	Design	Combustion Chamber	Model Number	Manufacturer
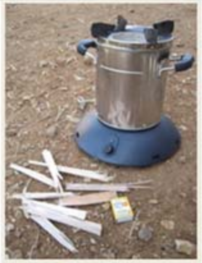	Philips	Electric fan-assisted gasifier	Ceramic	ACE (HD4012)	Philips African Clean Energy, Maseru, Lesotho, South Africa
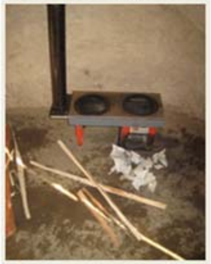	Prakti	Double pot rocket with chimney	Steel alloy	Leo	Prakti Pondicherry, India
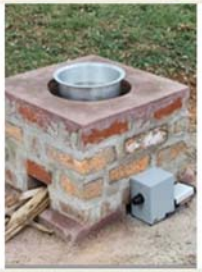	RTI TECA	Built-in mud rocket with thermal-powered fan	Brick/clay	Kenyan Upesi	Local artisans RTI International, Research Triangle Park, NC, USA
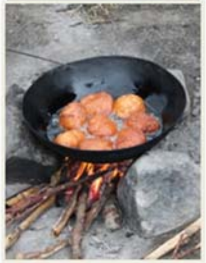	3 stone fire (TCS)	Stones	None	–	–

Table S2. Themes and codes.

**Stove Characteristics**: general preferences; fuel opening; versatility; efficiency; fit to kitchen; cleanliness; cooking speed; comfort; durability; ease of use, heat adjustment, ease of lighting, pot stability; knowledge of alternative stoves, fuels, experience of other stoves; local food preparation; multiple stove use; general concerns; appearance; patterns of use; placing; potential improvements; safety; smoke; stove maintenance; stove supervision, constant supervision, retaining a flame; taste of food; size combustion chamber;
**Fuel issues:** biomass availability; biomass collection; biomass quality; biomass quantity; ease of use; size of biomass; multiple fuel use, charcoal, kerosene
**Health:** child health; smoke, intensity/regularity, perceived hazards, burns, symptoms (description); smoke; maternal health, smoke, intensity/regularity, perceived hazards, burns, symptoms (description)

© 2016 by the authors; licensee MDPI, Basel, Switzerland. This article is an open access article distributed under the terms and conditions of the Creative Commons by Attribution (CC-BY) license (http://creativecommons.org/licenses/by/4.0/).
